# Supplementary material for: Complexome profiling of the Chlamydomonas psb28 mutant reveals TEF5 as an early PSII assembly factor
Source: Plant Cell. 2025 Mar 17;37(6):koaf055. doi: 10.1093/plcell/koaf055 (PMC12164590; doi:10.1093/plcell/koaf055)
Supplement: koaf055_Supplementary_Data [file koaf055_supplementary_data.zip › psb28_interactive.zip!/plots/intensity/qualityPlots/peptides/CPLD36_Cre08.g376450.t1.2.html]

### Cre08.g376450.t1.2

Trivial name: CPLD36  
SeqCoverage [median]: 14.90  
Mass: 15.09 kDa  
  
 

| Sequence | MissedCleavage | Mass [Da] | wtIntensity | muIntensity | Score |
| --- | --- | --- | --- | --- | --- |
| ADAAAEEAKAEAAAAPEPSK | 1 | 1896.91 | 0.00 | 2821.01 | 100.27 |
| KADAAAEEAKAEAAAAPEPSK | 2 | 2025.00 | 1676.84 | 5318.13 | 66.64 |
|  |  |  | 1676.8 | 8139.1 |  |
